# Monitoring trends in socioeconomic health inequalities: it matters how you measure

**DOI:** 10.1186/1471-2458-8-66

**Published:** 2008-02-20

**Authors:** Young-Ho Khang, Sung-Cheol Yun, John W Lynch

**Affiliations:** 1 Department of Preventive Medicine, University of Ulsan College of Medicine, Seoul, Korea; 2 Division of Biostatistics, Center for Medical Research and Information & Department of Preventive Medicine, University of Ulsan College of Medicine, Seoul, Korea; 3 Department of Epidemiology, Biostatistics and Occupational Health, McGill University, Montreal, Canada

## Abstract

**Background:**

Odds ratio (OR), a relative measure for health inequality, has frequently been used in prior studies for presenting inequality trends in health and health behaviors. Since OR is not a good approximation of prevalence ratio (PR) when the outcome prevalence is quite high, an important problem may arise when OR trends are used in data in which the outcome variable (e.g., smoking or ill-health) is of relatively high prevalence and varies significantly over time. This study is to compare time trends of odds ratio (OR) and prevalence ratio (PR) for examining time trends in socioeconomic inequality in smoking.

**Methods:**

A total of 147,805 subjects (71,793 men and 76,017 women) aged 25–64 from three Social Statistics Surveys of Korea from 1999 to 2006 were analyzed. Socioeconomic position indicators were occupational class and education.

**Results:**

While there were no significant p values for trend in ORs of occupational class among men, trends for PRs were significant. In women, p values for OR trends were similar to those for PR trends. In males, RII by log-binomial regression showed a significant increasing tendency while RII by logistic regression was stable between years. In females, trends of RIIs by logistic regression and log-binomial regression produced a similar level of p values.

**Conclusion:**

Different methods of measuring trends in socioeconomic health inequalities may lead to different conclusions about whether relative inequalities are increasing or decreasing. Trends in ORs may overstate or understate trends in relative inequality in health when the outcome is of relatively high prevalence and that prevalence varies significantly with time.

## Background

Monitoring the extent of socioeconomic health inequality over time is an essential element in policies aimed at reducing health inequalities. Various measures have been suggested and used for measuring relative magnitude of health inequality over time [1-3]. Odds ratio (OR), a relative measure for health inequality, has frequently been used in prior studies for presenting inequality trends in health and health behaviors [4-13] including ours [14,15]. Although OR is a good measure of association and can be a relative measure of health inequality, an important problem may arise when OR trends are used in data in which the outcome variable (e.g., smoking or ill-health) is of relatively high prevalence (e.g., > 10%) and varies significantly over time. As previously shown in several studies [16-21], odds exponentially increase as probability (outcome prevalence in cross-sectional data) increases and OR become greater compared to PR as outcome prevalence increases. Because of this nature of OR against PR, time trends of OR would be different from time trends of PR when outcome is of high prevalence and varies with time.

Table 1 represents a hypothetical example of this difference in time trends. If smoking rates in Time 1 are 75% and 60% for low and high social class respectively and there is no other confounder, PR is 1.25 (= 0.75/0.60) while OR is 2.00 (= 0.75/[1-0.75] ÷ 0.6/[1-0.6]). If smoking rates at Time 2 become 60% and 43% for low and high class respectively, OR becomes slightly smaller (1.99 = 0.6/[1-0.6] ÷ 0.43/[1-0.43]) despite increasing magnitudes of PR (1.40 = 0.6/0.43). This example demonstrates that OR trends may lead to a biased conclusion (no increase in relative inequality) when other relative measures of health inequalities (PR) indicate different results. This type of discrepancy can occur when we use other relative health inequality measures based on logistic regression, such as relative index of inequalities (RII). Therefore, the purpose of this study was to further explore this discrepancy by comparing time trends of OR and PR for presenting a possibility of discrepancy in time trends by two different relative health inequality measures in a nationally representative sample of South Korea.

**Table 1:** Hypothetical example* of trends of odds ratio and prevalence ratio by social class (low versus high class)

	Time 1		Time 2	
		
	Low class	High class	Low class	High class
Outcome prevalence (P)	0.75	0.60	0.60	0.43
Odds, P/(1-P)	3.00	1.50	1.50	0.75
OR^†^	2.00		1.99	
PR^‡^	1.25		1.40	

OR = odds ratio; PR = prevalence ratio.

* This example can be replicated when there is no other confounding variable.

^†^OR (odds ratio) was calculated by P_low class_/(1--P_low class_) ÷ P_high class_/(1--P _high class_).

^‡ ^PR (prevalence ratio) was calculated by P_lowclass _÷ P_high class_.

## Methods

### Data sources and study subjects

Data analyzed for this study were derived from the Social Statistics Survey conducted by the Korea National Statistical Office. These data are generated from face-to-face interviews conducted nationally for randomly selected households. Sections regarding health are included on the survey once every 3–4 years. Three rounds of publicly available Social Statistics Survey data (1999, 2003, and 2006) were used in this study. Non-response rates for these surveys were low (1.8% in 1999, 3.9% in 2003, and 1.6% in 2006). Data included 147,805 subjects (71,793 men, 76,017 women) aged 25–64.

### SEP indicators

Education and occupational class were used as indicators of socioeconomic position (SEP). Education levels were grouped into three categories (middle school or less, high school, and college or higher). Occupations in this study were based on the South Korean standard for classifying occupation, derived from the International Standard Classification of Occupation of the International Labor Organization [22]. Occupational class categories of on-manual vs. manual were employed [23]. Those who were not in the labor market (unemployed, retired, students and homemakers) were categorized as others. Non-manual occupations included managers, professionals, technicians, and clerks while manual occupations included service and sales workers, agricultural and fishery workers, craft and related trade workers, plant and machine operators and assemblers, and elementary occupations. Personal occupation was used for both men and women to define occupational class. Adults less than 25 years of age or those 65+ were also not included in the analysis as most of them were economically inactive.

### Smoking

The outcome variable for this study was current cigarette smoking measured by the question "Do you smoke tobacco now?" ("Yes, I smoke," "I smoked before but I quit smoking," "I never smoked"). The "Yes, I smoke" response was treated as a current smoker. Questions about smoking were consistent over the three waves of the Social Statistics Survey.

### Statistical analysis

All analyses were performed separately for men and women. We used absolute and relative measures to assess socioeconomic differentials in smoking rates. Age-adjusted rates were used as absolute measure. Education and occupation-specific smoking rates were calculated for 5-year age groups in each wave of the Social Statistics Survey data. These rates were directly standardized to 5-year age groups, using the age distribution of the 2005 South Korean census population. Confidence intervals (CI) of these age-standardized smoking rates were estimated, assuming a Poisson distribution of cases. Relative measures included the OR and RII computed by logistic regression and PR and RII estimated by log-binomial regression using PROC GENMOD of SAS statistical software (SAS Institute, Inc., Cary, North Carolina). Poisson regression is recommended for use in model fitting, when the log-binomial regression model does not converge. However, log-binomial regression estimates are more efficient when compared with the Poisson maximum likelihood estimators [24]. The RII measure, a relative measure for educational inequality in smoking, was needed to assess the summary effect of ordered SEP indicators and to take into account changes in the size of groups that are compared [1]. The RII has been used extensively in studies on trends in socioeconomic inequalities in health [14] and health behaviors, including smoking [5,15,25,26]. A relative educational position indicator was computed to calculate the RII. This indicator is a value between 0 and 1, assigned by calculating the mid-point of the relative position in the cumulative population distribution in each educational group, and was entered as an independent variable in the logistic regression and log-binomial regression. The RII by logistic regression is the odds of current smoking at the lowest end of the educational hierarchy as compared with the odds of current smoking at the very top of the educational hierarchy. By contrast, the RII by log-binomial regression is the prevalence ratio between two ends of educational hierarchy. Trends of OR, PR, and RII were estimated by examining the p value for an interaction term of SEP indicators and the variables that identified the year of the data in the model.

## Results

Table 2 presents calendar year- and gender-specific numbers of study subjects and crude smoking rate by education and occupational class. Educational levels for both genders increased by year and indicated the need for a health inequality measure such as RII for comparison of socioeconomic inequalities over time. However, the percentage of each occupational group did not vary significantly with year. Table 2 also reveals a rapid decrease in the crude smoking rate in men and socioeconomic differences in the crude rate among both genders.

**Table 2:** Calendar year- and gender-specific numbers of study subjects and crude smoking rate (%) by education and occupational class: 147,805 South Korean men and women aged 25–64 from 1999, 2003, 2006 Social Statistics Survey

	1999		2003		2006	
			
	N (%)	Crude smoking rate	N (%)	Crude smoking rate	N (%)	Crude smoking rate
**Men (total)**	23896 (100.0)	69.9	24495 (100.0)	58.7	23402 (100.0)	55.4
Occupational class						
Non-manual	6224 (26.1)	64.0	7215 (29.5)	52.8	7017 (30.0)	48.9
Manual	14149 (59.2)	73.4	13992 (57.1)	62.2	13062 (55.8)	59.3
Others	3523 (14.7)	66.4	3288 (13.4)	56.6	3323 (14.2)	54.2
Education						
College or higher	7437 (31.1)	64.1	9047 (36.9)	53.5	9425 (40.3)	50.9
High school	10371 (43.4)	74.2	10182 (41.6)	63.7	9476 (40.5)	60.5
Middle school or less	6088 (25.5)	69.7	5266 (21.5)	58.1	4501 (19.2)	54.4

**Women (total)**	24669 (100.0)	3.2	26121 (100.0)	2.9	25222 (100.0)	3.2
Occupational class						
Non-manual	2716 (11.0)	1.8	3950 (15.1)	1.4	4574 (18.1)	1.7
Manual	11212 (45.5)	4.1	10789 (41.3)	3.4	10132 (40.2)	4.2
Others	10741 (43.5)	2.6	11382 (43.6)	3.0	10516 (41.7)	3.0
Education						
College or higher	4080 (16.5)	1.5	6033 (23.1)	1.4	6829 (27.1)	1.6
High school	9762 (39.6)	2.7	10732 (41.1)	3.2	10462 (41.5)	3.7
Middle school or less	10827 (43.9)	4.2	9356 (35.8)	3.6	7931 (31.4)	4.0

As presented in Table 3, age-standardized prevalence rates of current smoking decreased in men aged 25–64 between 1999 and 2006. However, smoking rates among women aged 20–64 did not decrease. Table 3 shows that differences in age-standardized smoking rates were statistically significant between the college or higher and middle or less education groups and between non-manual and manual occupational class. This was true for men and women and true for all the years considered. Those differences, an absolute inequality measure, increased between 1999 and 2006. For example, prevalence difference in men between non-manual and manual occupational class was 7.9% in 1999 (73.6% minus 61.7%) but increased to 13.3% in 2006 (61.0% minus 47.7%). Absolute gaps in age-standardized smoking rates between the college or higher and middle school or less education groups also increased from 12.8% in 1999 to 15.7% in 2006 among men aged 25–64. These increases in socioeconomic inequality in smoking prevalence were also found in women.

**Table 3:** Age-standardized prevalence rates (95% CI), OR, PR, and RII of current cigarette smoking by calendar year, gender, and socioeconomic position indicators: 147,805 South Korean men and women aged 25–64 from 1999, 2003, 2006 Social Statistics Survey

	1999	2003	2006	p for trend
Men	69.9 (68.8–70.9)	58.9 (57.9–59.9)	56.1 (55.1–57.0)	
Occupational class				
Non-manual	61.7 (59.7–63.8)	50.7 (48.9–52.4)	47.7 (46.0–49.3)	
Manual	73.6 (72.2–75.0)	63.2 (61.9–64.6)	61.0 (59.6–62.4)	
Others	70.6 (67.3–73.9)	61.3 (58.2–64.4)	59.2 (56.1–62.3)	
OR (95% CI) of manual vs. non-manual	1.72 (1.62–1.84)	1.67 (1.57–1.77)	1.71 (1.61–1.82)	0.890
OR (95% CI) of others vs. non-manual	1.28 (1.17–1.40)	1.35 (1.24–1.47)	1.38 (1.27–1.51)	0.202
PR (95% CI) of manual vs. non-manual	1.17 (1.15–1.20)	1.23 (1.20–1.26)	1.27 (1.23–1.30)	< 0.0001
PR (95% CI) of others vs. non-manual	1.06 (1.03–1.09)	1.10 (1.07–1.15)	1.13 (1.08–1.17)	0.009
Education				
College or higher	61.2 (59.2–63.1)	50.4 (48.9–52.0)	48.5 (47.0–50.0)	
High school	72.5 (70.8–74.2)	63.1 (61.5–64.6)	61.5 (59.8–63.1)	
Middle school or less	74.0 (70.7–77.2)	65.5 (60.9–70.1)	64.2 (58.3–70.0)	
RII (95% CI) by logistic regression	2.69 (2.41–3.00)	2.82 (2.55–3.12)	2.83 (2.55–3.13)	0.524
RII (95% CI) by log-binomial regression	1.31 (1.27–1.35)	1.51 (1.46–1.57)	1.58 (1.51–1.64)	< 0.0001

Women	3.1 (2.9–3.4)	2.9 (2.7–3.1)	3.2 (3.0–3.4)	
Occupational class				
Non-manual	1.9 (0.9–2.9)	1.3 (0.9–1.8)	1.5 (1.1–1.9)	
Manual	4.1 (3.7–4.5)	3.9 (3.4–4.4)	4.6 (4.1–5.2)	
Others	2.6 (2.3–3.0)	3.0 (2.7–3.3)	3.0 (2.6–3.3)	
OR (95% CI) of manual vs. non-manual	1.74 (1.28–2.37)	2.28 (1.70–3.05)	2.61 (2.02–3.38)	0.071
OR (95% CI) of others vs. non-manual	1.19 (0.87–1.63)	2.01 (1.51–2.69)	1.84 (1.42–2.38)	0.058
PR (95% CI) of manual vs. non-manual	1.71 (1.26–2.32)	2.24 (1.68–2.98)	2.55 (1.98–3.27)	0.072
PR (95% CI) of others vs. non-manual	1.20 (0.88–1.63)	1.99 (1.49–2.64)	1.81(1.41–2.33)	0.063
Education				
College or higher	1.9 (1.1–2.7)	1.6 (1.1–2.2)	1.6 (1.2–2.0)	
High school	2.8 (2.4–3.2)	3.2 (2.9–3.6)	3.9 (3.5–4.4)	
Middle school or less	3.8 (3.2–4.3)	4.1 (3.0–5.2)	7.5 (4.5–10.5)	
RII (95% CI) by logistic regression	2.28 (1.63–3.19)	3.28 (2.38–4.53)	3.52 (2.60–4.78)	0.067
RII (95% CI) by log-binomial regression	2.22 (1.61–3.08)	3.17 (2.32–4.34)	3.39 (2.52–4.56)	0.066

CI = confidence intervals; OR = odds ratio; PR = prevalence ratio; RII = relative index of inequality.

Table 3 shows OR and PR that measure relative levels of occupational inequalities in smoking between 1999 and 2006. Differences between OR and PR values were significant among men with high rates of smoking while minimal differences were found among women with low smoking rates (less than 5%). In men, OR for those in the manual occupational class showed a stable trend by year. OR was 1.72 in 1999 but slightly decreased to 1.67 in 2003 and finally returned to the 1999 level in 2006 (OR = 1.71). However, PR showed a consistent increasing tendency: 1.17 in 1999, 1.23 in 2003, and 1.27 in 2006. While there were no significant p values for trend in ORs for those in the manual work and others group, trends for PRs were significant for both manual workers and others group. By contrast, in women, p values for OR trends were similar to those for PR trends.

Table 3 also shows RIIs for education estimated by logistic regression and log-binomial regression. Analysis results were similar to those regarding OR and PR. In men, logistic regression RII values were much greater than values by log-binomial regression. Results for men in RII by log-binomial regression also showed a significant increasing tendency while RII by logistic regression was stable between years. However, in women, trends of RIIs by logistic regression and log-binomial regression produced similar p values for time trends.

## Discussion

Smoking rates in South Korean men decreased between 1999 and 2006 but were still very high (> 50%) while smoking rates in women were very low (< 5%) but did not decrease. This finding is an extension of a previous analysis [15] and generally agrees with previous studies using different sources of South Korean data [26,27].

Results of this study demonstrate that differences in the conclusions can be drawn about trends in socioeconomic inequality in smoking, depending on whether trends in OR and PR were used. This was also true for RIIs estimated by logistic regression and log-binomial regression. Although PR and RII by log-binomial regression as well as absolute differences in age-adjusted prevalence of current smoking showed a widening socioeconomic inequality, OR and RII by logistic regression presented no increase in relative inequalities. The discrepancy was evident for men whose smoking prevalence was quite high (over 50%). This is because OR is not a good approximation of PR and thus can be misleading in measuring relative socioeconomic health inequalities when the outcome prevalence is high (> 10%). However, OR and RII by logistic regression were not discrepant from PR and RII by log-binomial regression for women because of the "rare disease assumption" (i.e. less than 10% of women smoked).

Including us [14,15], many researchers have used OR trends as a measure for trends in relative socioeconomic inequality in health when several rounds of data with a dichotomous outcome variable were analyzed [7,8,10-13]. In cases with ordered SEP indicators such as education and income, RII by logistic regression has been used [4-6,9,25,26]. However, it should be noted that use of these measures does not necessarily produce a biased result on relative socioeconomic inequality in health. When the outcome is rare, OR and RII by logistic regression can be a reliable relative measure for monitoring health inequality as our research finding in women shows. However, if the outcome prevalence is high and varies significantly over time, the chance for a discrepancy between trends of OR and PR become greater. This is due to the exponential nature of odds against prevalence [16-21]. If prevalence of the common outcome rises with year while absolute socioeconomic difference in prevalence gets smaller, PR by SEP becomes smaller with year. In this case, OR by SEP may be stable over time or even increase since OR disproportionately increases as prevalence rises. By contrast, if the prevalence of a common outcome decreases with increasing trends in absolute prevalence difference, the PR trend shows a widening relative inequality while the OR trend may be stable or even decrease with time. This latter case was demonstrated for men in this study. These results can be seen in previous studies. For example, in a British study examining changes in the social distribution of cardiovascular risk factors over time (p. 810) [5], the age-standardized percentage of those drinking high fat milk was 81.9% for high professional and managerial workers and 91.1% for semi- and non-skilled workers; a 9.2% difference. Over time, the percentage plummeted to 27.7% for high professional and managerial workers and 51.1% for semi- and non-skilled workers; a 23.4% difference. According to our calculation based on the age-adjusted percentage of those drinking high fat milk, PR increased from 1.11 (= 91.1%/81.9%) to 1.84 (51.1%/27.7%). However, RII in the study [5] tended to decrease from 3.87 to 3.66.

When outcome prevalence is high but does not vary much with time, it would not be expected that OR trends would be discrepant from PR trends. However, even in this case, conclusions based on the small difference in OR should be cautioned. For example, in a recent international comparison study on socioeconomic inequality trends in self-assessed health [10], PR of 'fair/poor' self-assessed health between the lowest versus highest educational level among Finnish men showed an increasing tendency from 1.88 (= 48.8%/25.9%) in the 1980s to 1.91 (= 45.7%/23.9%) in the 1990s (p. 300), according to our calculation based on the age-adjusted prevalence. However, OR presented in the study indicated a decreasing trend from 3.15 in 1980s to 2.99 in 1990s (p. 301), although a definitive conclusion was not given regarding trends of educational inequalities in self-assessed health among Finnish men.

## Conclusion

In summary, this study compared time trends of OR and PR in smoking trends of South Korean men and presented different results. Socioeconomic differences in age-adjusted prevalence of smoking, an absolute measure for health inequalities, increased with year. OR and RII by logistic regression showed stable trends in socioeconomic inequality in smoking while PR and RII by log-binomial regression presented clear increasing trends. This was evident in men whose smoking rate was quite high and varied significantly with year. Results of this study show that using OR trends may lead to a different conclusion regarding trends of relative inequality in health when the outcome is of relatively high prevalence and varies significantly with time. This is significant because OR trends have been widely used to examine socioeconomic health inequalities over time as binary outcome data with a cross-sectional design can be one of the most prevalent source for monitoring health inequality. As PR can be easily computed [24], diverting a researcher's use of relative health inequality measure from OR to PR is required when prevalence is relatively high.

## Abbreviations

CI = confidence interval; OR = odds ratio; PR = prevalence ratio; RII = relative index of inequality; SEP = socioeconomic position

## Competing interests

The author(s) declare that they have no competing interests.

## Authors' contributions

YHK conceived and designed the study with advice from SCY and JWL. YHK analyzed with assistance of SCY and interpreted the data and drafted the paper. SCY and JWL interpreted the data and critically revised the draft of the paper. All authors read and approved the final manuscript.

## Pre-publication history

The pre-publication history for this paper can be accessed here:


